# Extranodal NK/T-cell lymphoma, nasal type–a case report and a review of the literature

**DOI:** 10.3389/fonc.2025.1560442

**Published:** 2025-05-14

**Authors:** Yu Zhan, Na Sun

**Affiliations:** Department of Otorhinolaryngology, Guanghua Hospital Affiliated to Shanghai University of Traditional Chinese Medicine, Shanghai, China

**Keywords:** lymphoma, T-cell, hemolymphatic dissemination, nasal, EBV, episodic

## Abstract

Nasal-type extranodal NK/T-cell lymphoma (ENKTCL) is a unique type of mature NK/T-cell lymphoma that is closely related to the EBV virus (EBV). In most cases, ENKTCL occurs in the nasal cavity and other parts of the upper respiratory and digestive tract, and its clinical presentation is usually unremarkable, making it difficult to early diagnosis. We report a case of NK/T-cell lymphoma and its main clinical features and cytopathological characteristics.

## Introduction

Nasal-type extranodal NK/T-cell lymphoma (ENKTCL) is a unique type of mature NK/T-cell lymphoma that is closely related to the EBV virus (EBV) (1). It is more common to get in Asian populations and Native Americans than in Western populations. In most cases, ENKTCL occurs in the nasal cavity and other parts of the upper respiratory and digestive tract, and its clinical presentation is usually unremarkable, making it difficult to early diagnosis. However, the disease is aggressive with a rapidly progressive clinical progress, which causing patients had a poor level of prognosis and limited Expectations for survival (2). In this paper, we report a case of NK/T-cell lymphoma that was diagnosed early by examination of cytologic and histologic specimens and got controlled. In addition, the main clinical features and cytopathological characteristics of this disease of the hemolymphatic system are reviewed.

## Case report

### Material and methods

#### Patient consent

**Figure 1:**
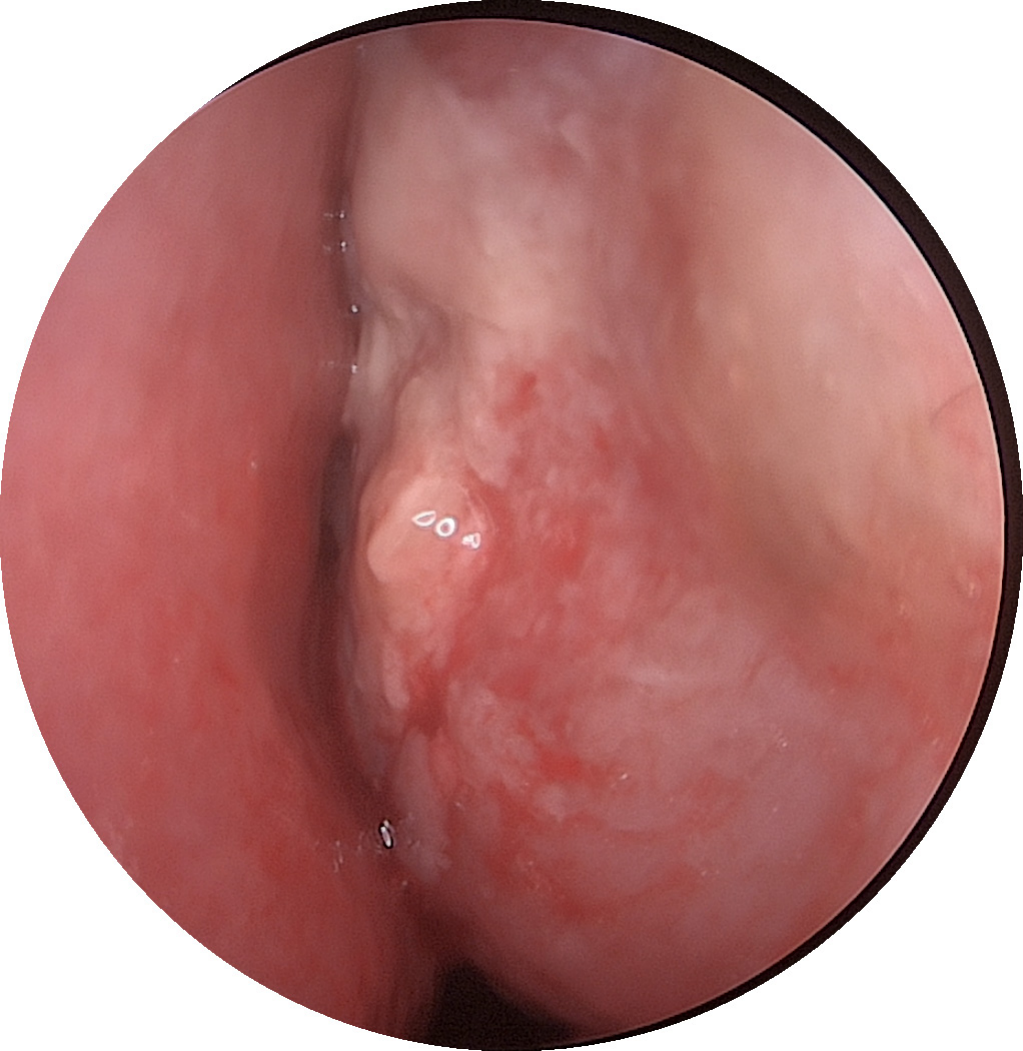
An electronic nasal endoscopy was performed:the left inferior turbinate was hypertrophied with mucosal erosion at the anterior end.

**Figure 2:**
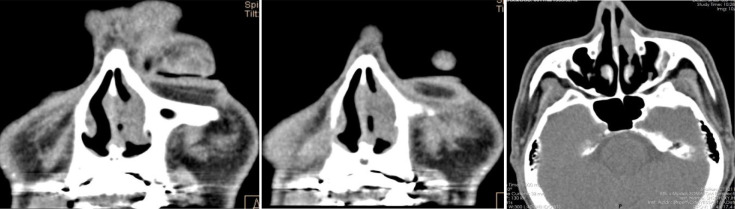
After operation PET-CT show that the soft tissue shadow of the left nasal cavity and the anterior part of the nasal septum is slightly thickened, and the FDG metabolism is increased, which may be consistent with lymphoma. No lymphoma metastasis was seen.

**Figure 3:**
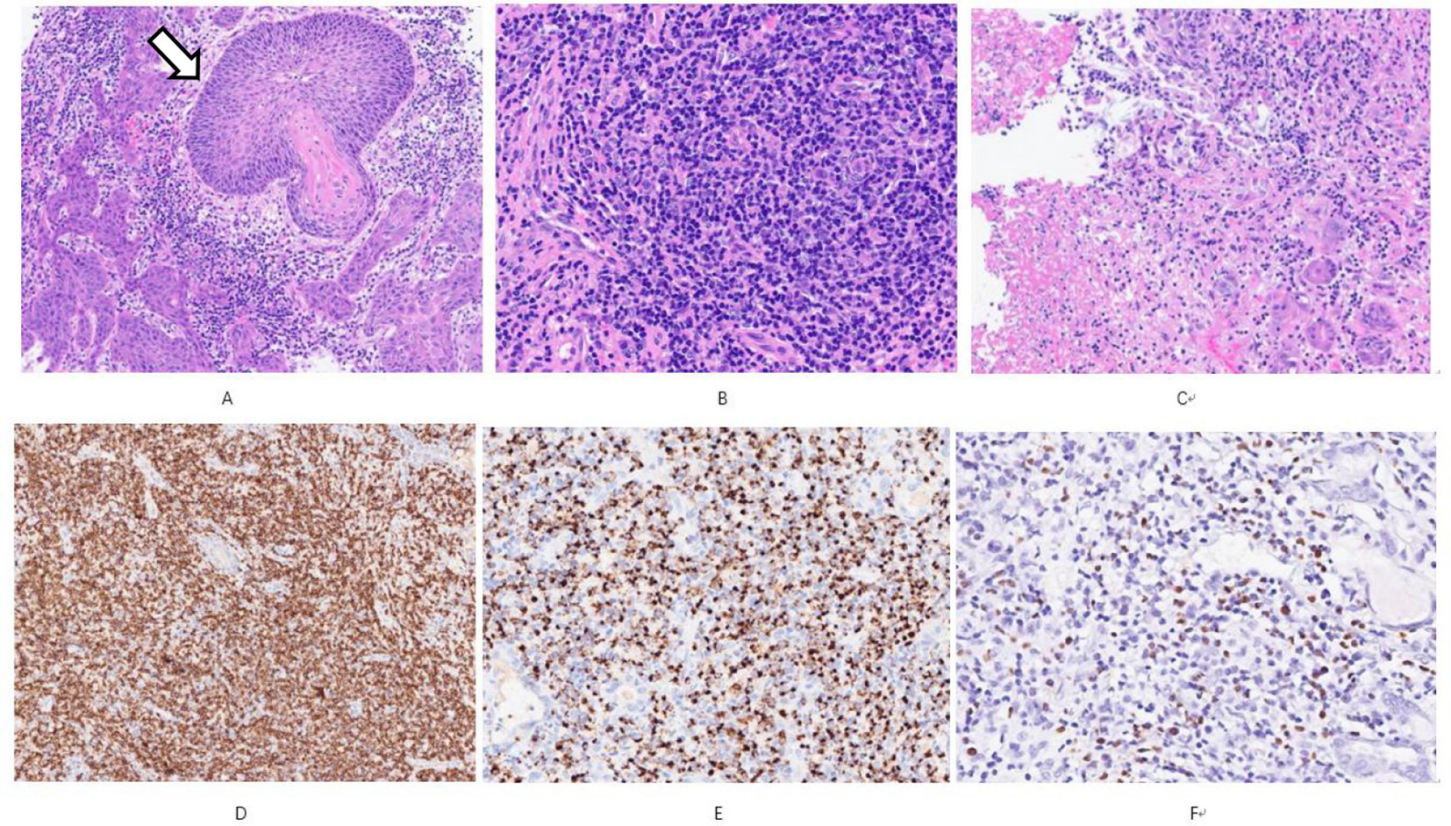
**(A)** shows obvious pseudoepitheliomatous hyperplasia (open arrows), resembling squamous cell carcinoma HE Low magnification; **(B)** the tumor is predominantly small cells mixed with more plasma cells and a few eosinophils HE Medium magnification; **(C)** coagulative necrosis is seen in the lesion HE Low magnification; **(D)** shows diffuse CD56 positivity Immunohistochemistry Low magnification; **(E)** shows Granzyme B positivity Immunohistochemistry Medium magnification; **(F)** shows EBER positivity *In situ* hybridization Medium magnification.

**Figure 4:**
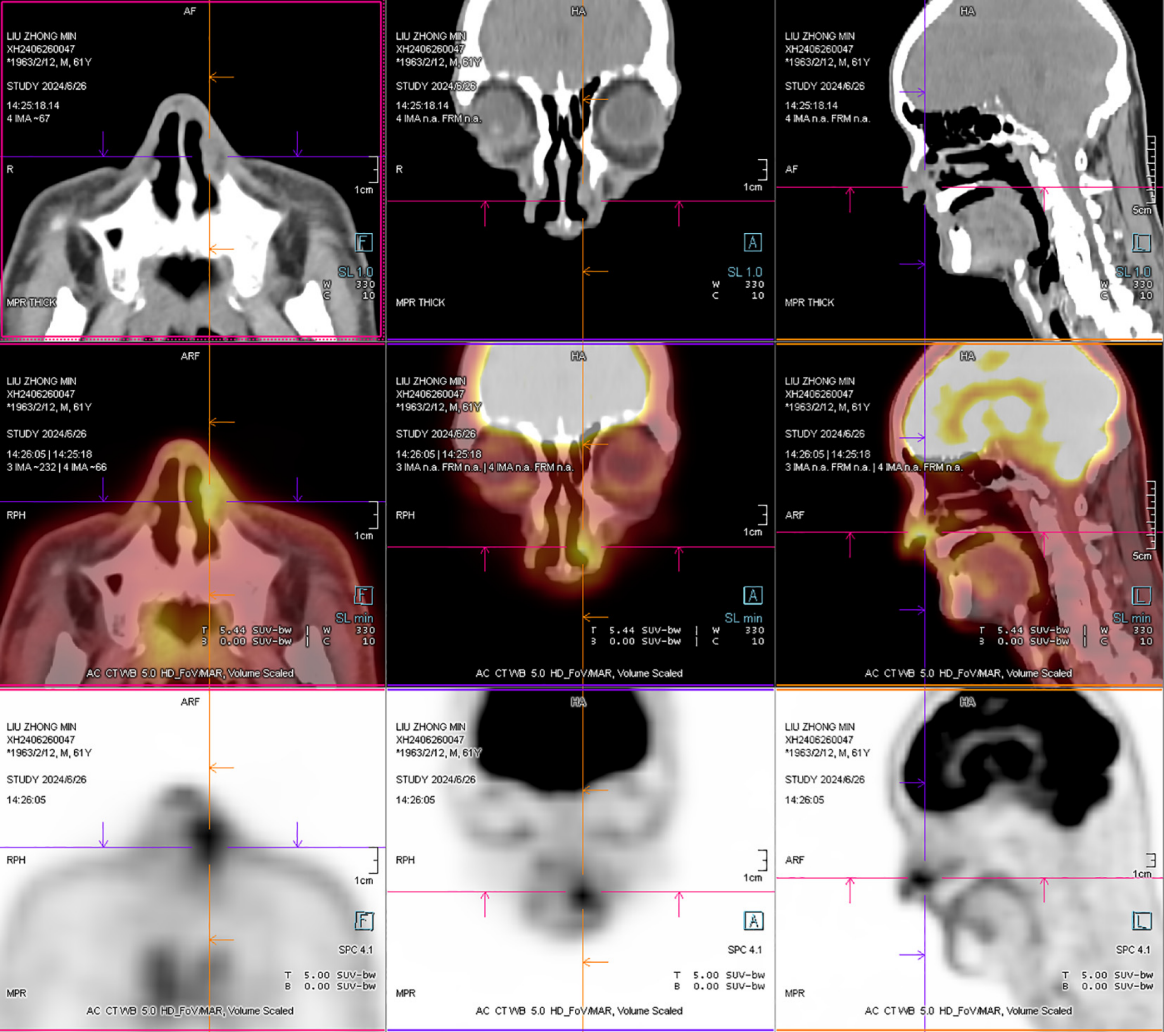
After operation PET-CT show that the soft tissue shadow of the left nasal cavity and the anterior part of the nasal septum is slightly thickened, and the FDG metabolism is increased, which may be consistent with lymphoma (open arrows). No lymphoma metastasis was seen. (The PETCT whole-body scan examination revealed no FDG hypermetabolic signals in other parts except for the lesion in the nasal cavity. Therefore, metastasis in other parts can be excluded.

**Figure 5:**
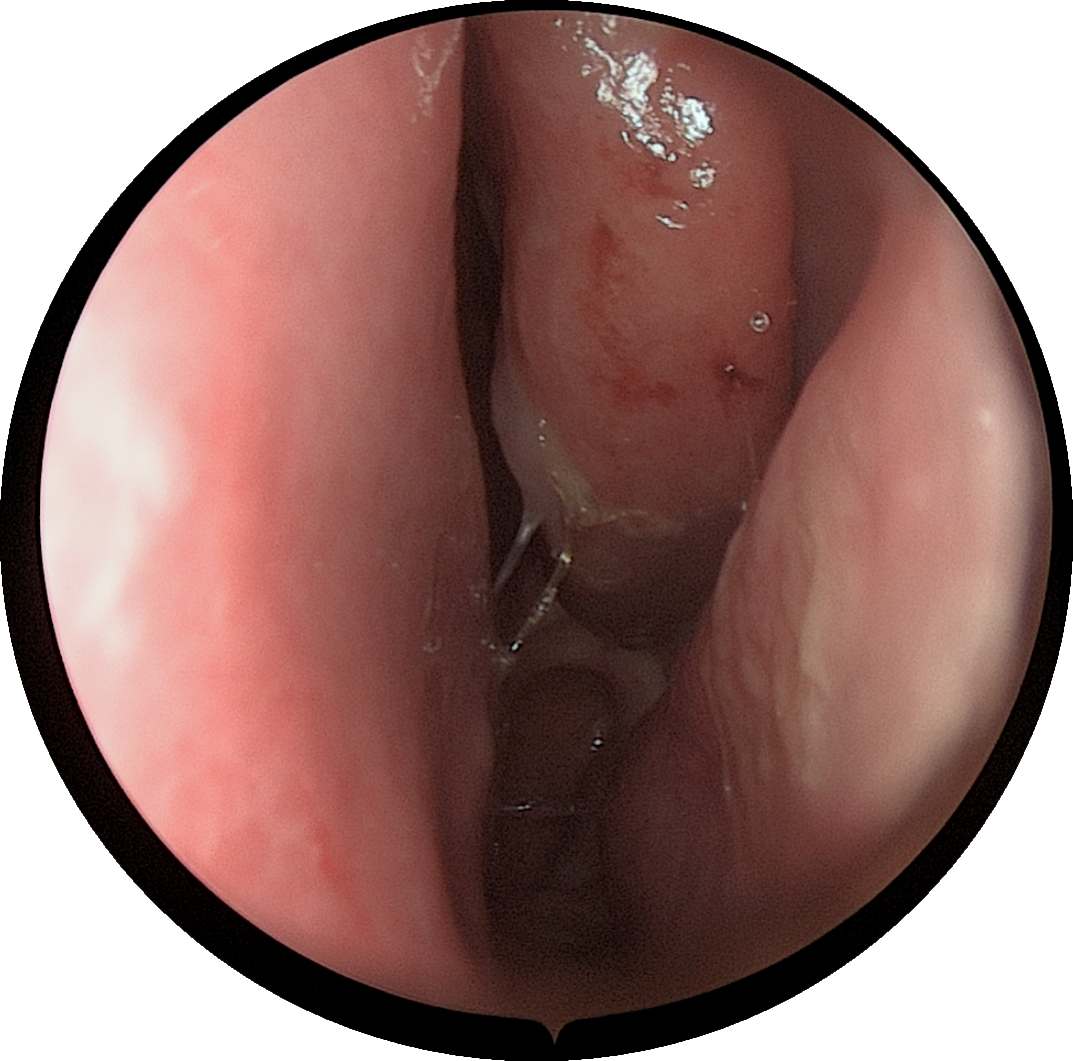
Nasal endoscopy showed smooth nasal mucosa after six months of surgery.

A 61-year-old male patient, presented with persistent nasal congestion and runny nose for 4 years, accompanied by episodic epistaxis without trigger. An electronic rhinoscopy under surface anesthesia was performed: the left inferior turbinate was hypertrophied with mucosal erosion at the anterior end, the middle nasal passages were clear bilaterally, and the mucous membrane of the nasopharynx was smooth (**Figure 1**), and a sinus CT examination was performed (**Figure 2**). Based on clinical suspicion of possible malignancy, left nasal inferior turbinate and neoplasm resection was performed. Pathologic examination showed diffuse mucosal necrosis with granulation tissue formation and dense cellular infiltration. Atypical lymphocytes were observed in the diseased tissue with variable size, irregular and distinctive nuclear shape, and pale or clear cytoplasm. Larger atypical lymphocytes were also found, accompanied by significant apoptosis and scattered mitotic figures. In addition, plastic cells showed a vasculocentric growth pattern with vascular destruction, mural fibrin-like necrosis and hemorrhage. The residual squamous epithelium showed pseudoepithelial hyperplasia (**Figures 3A–C**). Immunohistochemical testing showed that the atypical lymphocytes stained positive for CD56, granzyme B and CD3, whereas CD20 and cytokeratin (AE1/AE3) staining was negative. Most of the atypical lymphocytes showed a CD4-/CD8- phenotype. In addition, a mixed distribution of small CD4+ or CD8+ lymphocytes was seen in the background. Lymphoma was highly suspected, and EBER *in situ* hybridization was applied to detect EBV RNA-positive signals in a large number of atypical lymphocytes, supporting the diagnosis of EBV-associated lymphoma (**Figures 3D–F**). The diagnosis of NK/T-cell lymphoma (nasal type) was confirmed on the basis of histopathologic features and immunohistochemical results. PET-CT was performed to exclude the tumor spread, and no lymphoma metastasis was seen (**Figure 4**). The PETCT whole-body scan examination revealed no FDG hypermetabolic signals in other parts except for the lesion in the nasal cavity. Therefore, metastasis in other parts can be excluded. After surgery, the patient was treated with a radiotherapy regimen(Pemetase 2000 U/m² intramuscularly on day 1, gemcitabine 1000 mg/m² IV on days 1 and 8, and oxaliplatin 130 mg/m² IV on day 1 for 4 cycles; sequential radiotherapy (50–56 Gy) within 4 weeks after final chemotherapy) in the hematology department with regular follow-up. Nasal endoscopy after six months showed smooth nasal mucosa (**Figure 5**). The patient’s condition was stable, and no local tumor recurrence or metastasis was seen.

## Discussion

Natural killer (NK)/T-cell lymphoma was officially named extranodal NK/T-cell lymphoma, nasal type (ENKTCL) in the 2008 and 2016 World Health Organization (WHO) classifications (3, 4). The disease exhibits a wide range of histopathologic changes and is notably characterized by a pleomorphic infiltrate of small, medium, and large atypical lymphocytes, which often have irregular nuclei, prominent nucleoli, and clear or pale blue cytoplasm. In addition, the infiltrating lesions are often scattered with plasma cells, macrophages, and neutrophils, accompanied by varying degrees of necrosis. Typical histologic features also include vasocentric infiltration or vascular destructive infiltration, these pathologic manifestation were thought to be closely related to the aggressive nature of the disease (5).

ENKTCL usually has a poor prognosis, but the present case of an early-stage patient without metastasis suggests that paying attention to symptoms such as epistaxis, timely consultation for nasal endoscopy and tissue excision for pathologic examination for definitive diagnosis are the key tools to improve the patient’s prognosis (15).

Nasal mucosal manifestations (e.g., unilateral nasal congestion, recurrent epistaxis, mucosal erosions or necrosis) in early ENKTCL are often confused with rhinitis/sinusitis, leading to an average delay in diagnosis of 4–6 months. Typical endoscopic features include brittle hemorrhagic mucosa, pseudomembranous overlying membranes, or nodular changes, but the diagnosis is missed in about 50% of cases due to necrosis or inflammatory background masked by biopsy sampling. Key diagnostic clues are (i) symptoms not responding to anti-inflammatory therapy, (ii) EBER+ atypical lymphocytes presenting as a vasocentric infiltrate (even if CD56-), and (iii) imaging findings of mucosal thickening with early bone erosion. Improving early recognition requires deep multisite biopsies and routine testing of EBER in patients with persistent nasal symptoms to avoid misdiagnosis of infection or inflammatory disease (16).

Typical NK/T-cell lymphomas are characterized by a specific T-immunophenotype with immunohistochemical features that include positive staining for CD2, CD56, and cytoplasmic CD3ϵ, whereas surface CD3 is usually negative (6). In addition, markers such as CD43 and CD45RO are often positively expressed (7). In some cases, positive staining for CD30 is also observed (8). Although there may be variability in the immunoreactivity of LMP-1, almost all NK/T-cell lymphoma cells exhibit positive labeling for EBV-encoded small RNA (EBER) in *in situ* hybridization assays. This assay is now considered one of the most reliable means of confirming the diagnosis of NK/T-cell lymphoma (9).

The cytologic features of nasal-type NK/T-cell lymphomas have not been fully corroborated in the available literature, and their cytologic manifestations are widely diverse, ranging from predominantly small cells to predominantly large cells (10). On fine-needle aspiration (FNA) smears, a mixture of small benign lymphocytes with variable numbers of small to large atypical lymphocytes is usually observed, while plasma cells, eosinophils, and histiocytes may also be present. The appearance of the tumor cell population may vary significantly among individuals, but in most cases, medium-sized cells are seen that have granular chromatin, most present multiple small and inconspicuous nucleoli, and are accompanied by lightly stained cytoplasm (11). The nuclear heterogeneity of the tumor cells is striking, with irregularly shaped nuclei, including elongated, twisted, “banana-like” or “cucumber-like” features. In addition, “tongue-like” protrusions are often observed in the cytoplasm of the tumor cells, and this cytomorphological feature may be a useful clue for diagnosis (12).

However, there are significant difficulties in early diagnosis of nasal NK/T-cell lymphoma. First, because of the diverse cytologic manifestations of this lymphoma and the fact that early tumor cells may be predominantly small, these small cells lack typical heterogeneity and are easily confused with small lymphocytes in chronic inflammation or other reactive lesions. Second, tumors are often accompanied by significant necrosis and inflammatory cell infiltration, and these background features may mask the presence of tumor cells in FNA smears, making them difficult to identify. In addition, ENKTCL-NT usually has no specific clinical manifestations in the early stages, and patients with symptoms such as nasal congestion or rhinorrhea, discomfort with pus, or headache have atypical early symptoms, which can be easily misdiagnosed as common infectious or inflammatory diseases (13). Early CT manifestations are atypical, mostly soft tissue density shadow, with a certain degree of infiltration in the progressive stage. In this case, the patient showed persistent nasal congestion, intranasal crusting, and occasional blood in the snot, which was easy to be misdiagnosed as nasal mucosal erosion or hypertrophic rhinitis, and the left inferior turbinate was hypertrophic on admission, the surface was not smooth, and it was easy to bleed by touch, and there was localized erosion and necrosis on intraoperative exploration, which made us highly vigilant for the possibility of malignant tumors in the nasal cavity. The definitive diagnosis depended on histopathological analysis and immunohistochemistry. Tumor cell immunohistochemistry examination can be seen markers CD3 and granzyme B positive, NK series of tumors can be seen CD56 +, T series of tumors can be seen CD5 +, CD8 +, NK and T series of tumors there is no obvious clinicopathological difference, so clinical diagnosis and treatment cannot be separated from the two. There is no standardized and uniform treatment method for this disease, and clinical reports include radiotherapy, chemotherapy, radiochemotherapy (synchronous, sequential or “sandwich” method), stem cell transplantation, immunotherapy and targeted therapy, etc. However, the effects of different treatment methods are mostly different from each other. The effects of different treatments are mostly analyzed retrospectively, with different sample sizes, more bias factors, and lack of data support from prospective large-sample randomized controlled studies. In this case report, We choose PGemOx as the treatment, Compared with traditional regimens such as SMILE/DDGP, PGemOx (pembrolase + gemcitabine + oxaliplatin) has the characteristics of lower toxicity (no methotrexate/cisplatin), more convenient administration (can be done on an outpatient basis), and more synergistic radiotherapy (sensitization by gemcitabine) while maintaining similar efficacy, which is especially suitable for patients who are elderly, frail, or in need of combined radiotherapy. However, this article prefers to emphasize its early diagnostic skills and how ENT clinics can improve the diagnosis rate and enhance patients’ quality of life (17).

## Conclusion

Early and accurate pathological diagnosis and timely treatment of NK/T-cell lymphoma are essential to improve prognosis due to its typically aggressive behavior (14). Although the disease can be initially diagnosed by cytomorphology, the diverse and nonspecific presentations on conventional cytologic studies often make the diagnosis more difficult. A review of case series suggests that a combination of ancillary tests, such as FNA or body fluid cytology, can be effective in improving diagnostic accuracy. The diagnosis depends on the pathological analysis of the tissue removed during surgery. This case fits the typical clinicopathologic features of ENKTCL (prolonged nasal congestion/epistaxis, angiocentric growth, EBER+), but the 4-year duration of the disease is significantly longer than the average diagnostic delay and the limited-stage lesions responded well to treatment, suggesting that a better prognosis may be possible even with a longer history of the disease. Cellular material is key to the diagnosis, and a combination of immunocytochemical markers (e.g., CD56, granzyme B, EBER) and flow cytometry can further improve the sensitivity and specificity of the diagnosis. Comprehensive evaluation of the patient’s clinical features, cytologic and histologic manifestations, combined with immunophenotypic analysis, is the key to achieving a definitive diagnosis. Early pathologic diagnosis provides the basis for timely treatment, which helps to delay disease progression and improve survival. Therefore, doctors should fully combine cytology, histology and molecular pathology techniques to identify and confirm the diagnosis of this type of highly aggressive lymphoma, while strengthening multidisciplinary collaboration to explore more sensitive early diagnostic markers.

## Data Availability

The original contributions presented in the study are included in the article/supplementary material. Further inquiries can be directed to the corresponding author.
